# Optically induced diffraction gratings based on periodic modulation of linear and nonlinear effects for atom-light coupling quantum systems near plasmonic nanostructures

**DOI:** 10.1038/s41598-020-73587-y

**Published:** 2020-10-07

**Authors:** Azar Vafafard, Mostafa Sahrai, Vahid Siahpoush, Hamid Reza Hamedi, Seyyed Hossein Asadpour

**Affiliations:** 1 Faculty of Physics, University of Tabriz, Tabriz, Iran; 2 Institute of Theoretical Physics and Astronomy, Vilnius University, Saulėtekio 3, 10257 Vilnius, Lithuania; 3 Young Researchers and Elite Club, Central Tehran Branch, Islamic Azad University, Tehran, Iran; 4 Research Institute for Applied Physics and Astronomy, University of Tabriz, Tabriz, Iran

**Keywords:** Optics and photonics, Physics

## Abstract

We investigate the quantum linear and nonlinear effects in a novel five-level quantum system placed near a plasmonic nanostructure. Such a quantum scheme contains a double-V-type subsystem interacting with a weak probe field. The double-V-subsystem is then coupled to an excited state by a strong coupling field, which can be a position-dependent standing-wave field. We start by analyzing the first-order linear as well as the third and fifth order nonlinear terms of the probe susceptibility by systematically solving the equations for the matter-fields. When the quantum system is near the plasmonic nanostructure, the coherent control of linear and nonlinear susceptibilities becomes inevitable, leading to vanishing absorption effects and enhancing the nonlinearities. We also show that when the coupling light involves a standing-wave pattern, the periodic modulation of linear and nonlinear spectra results in an efficient scheme for the electromagnetically induced grating (EIG). In particular, the diffraction efficiency is influenced by changing the distance between the quantum system and plasmonic nanostructure. The proposed scheme may find potential applications in future nanoscale photonic devices.

## Introduction

During the recent decades, nonlinear optical properties of various atomic^1–4^ and other coherent media^5,6^ have been well studied due to quantum interference and coherence. As examples and owning to their wide range of applications in quantum technologies, one can evoke spontaneous emission^7,8^, population distribution^9^, large refractive index^10^, Kerr nonlinearity^11^, optical solitons^12^, and magneto optical rotation^13^. The quantum coherence and interference effects have been studied in other media by studying the optical susceptibilities^14,15^. For example, Dolgaleva et al.^16^ experimentally observed the nonlinear properties of mixture of Carbon disulfide (CS_2_) and fullerene C60.

On the other hand, there has been a great deal of interest on the effect of an environment on the emission properties of an emitter^17^. For instance, in an early study, Becchmann^18^ showed how the radiation resistance of an antenna could be affected by the integration of the Poynting vector over a surface enclosing the system.

Following Ref.^18^ many works have been proposed in terms of the imaginary part of the classical electromagnetically (EM) Green's function which led to creation of quantum interference^19,20^. It has been shown that the quantum interference between two spontaneous emission transitions can be greatly enhanced by using the left-handed materials^19^. The spontaneous emission of a two-level quantum system near left-handed slab as well as the contribution of the guided modes and the surface-plasmon polariton modes on the spontaneous decay of the atomic system have been also investigated in^20^.

It is known that the quantum nonlinear optical properties of a multi-level atomic system can be modified when the quantum systems are placed near plasmonic nanostructures^21–24^. A recent Review discusses on the complex quantum systems consisting of multiple photon and plasmon^25^. It has been shown that the photon-plasmon interactions play an important role on optical properties of multi particle system.

As a pioneering work, Yannopapas et al.^26^ suggested an efficient scheme for enhancement of quantum interference of two spontaneous emission channels in a three-level V-type atomic system near plasmonic nanostructure due to surface plasmon effect. Different proposals have been considered for controlling the optical properties of three or four-level quantum systems near metallic nanostructures afterward^27–30^. For instance, the coherent control of free-space spontaneous emission in a four-level quantum system has been studied in the presence of plasmonic nanostructure^28^. It has been shown that due to the interaction between surface plasmon and atomic transitions, the spontaneous emission spectrum of a four-level quantum emitter is strongly influenced by the distance between the quantum system and plasmonic nanostructure. Optical transparency and slow light have also been analyzed for a four-level quantum system near a plasmonic nanostructure^29^. Phase-sensitive optical properties of a four-level quantum system near plasmonic nanostructure have been studied by the same group^30^. It is shown that the absorption and dispersion of the quantum system for a weak probe and coupling lights becomes sensitive to the relative phase of applied fields. Therefore, by changing the relative phase, one can obtain gain without population inversion for both probe and coupling lights. The Kerr nonlinear behavior of a four-level quantum system interacting with only a weak probe light has also been discussed^31^, and realized that the nonlinear properties of the medium is impacted by changing the distance between the quantum system and plasmonic nanostructure. Recently, Carreno and coworkers investigated the optical response of a four-level double V-type quantum system near plasmonic nanostructure when the quantum system is interacting simultaneously with a probe and pump laser fields^32^. Because of the different coupling configuration for the pump/probe laser fields, significant absorption, gain without population inversion, and phase-dependent absorption curves have been observed.

By applying an intensity-dependent standing-wave field in an electromagnetically induced transparency (EIT) medium, the probe light propagating through the medium experiences periodic variation of absorption and refraction leading to the electromagnetically induced grating (EIG)^33^. This makes the medium acting as a Bragg or diffraction grating that causes the diffraction of probe light to high-order directions^34^. Several setups have been proposed for efficient control of EIG patterns in atomic or semiconductor quantum well nanostructures^35–40^. For example, two dimensional (2D) EIG in a double Λ-type atomic system was proposed utilizing incoherent pumping field^39^. It was realized that the absorption of the probe light could be vanished or amplified by using an incoherent pumping field. In this case, the refractivity of the medium enhances and the phase grating or gain-phase grating also appears. Hence, the probe light diffracts to high-order directions when propagating inside the medium.

Recently, an analysis has been carried on the plasmon-induced phase grating in a four-level quantum system near plasmonic nanostructure^41^. It was found that due to the presence of plasmonic nanostructure, the medium becomes phase-dependent. Therefore, the energy can be transferred from zero-order to high-order grating by changing the relative phase of applied fields.

The effect of tunneling induced transparency on diffraction efficiency of a weak probe light has been also studied in a multiple quantum well driving by a 2D standing-wave pattern^42^. It is realized that by adjusting the third and fifth order optical susceptibilities, the probe energy can transfer from zero order to high orders of gratings. Moreover, it is found that in off-resonance conditions of coupling field, the enhanced nonlinear susceptibilities have essential role for transferring the probe energy from zero to high orders of diffraction.

The current study extends the previous works to a five-level quantum system near plasmonic nanostructure through coherently adjusting nonlinear parts of susceptibility (up to fifth order). A large high-order nonlinear response can be achieved due to the presence of the plasmonic nanostructure near the proposed quantum system. Such a property could be useful for creating EIG with new control mechanism. The efficiency of the diffraction can be manipulated by changing the distance between the atomic system and plasmonic nanostructure. Competition between the linear and nonlinear susceptibilities enables the switching between amplitude and phase gratings in the proposed EIG. Such a new switching mechanism has been introduced by the presented study.

The proposed atom-light coupling here contains a double V-type atomic subsystem which interacts by a weak probe light and simultaneously couples to an excited state by a coupling light with the standing-wave pattern. Therefore, the susceptibility of the medium can be expanded to higher orders of coupling field. By using the Taylor series, we extend the susceptibility up to fifth-order and then discuss linear and nonlinear properties as well as the EIG patterns of the weak probe light for different distances between quantum system and plasmonic nanostructure. It is found that for different spatial distances between the quantum system and plasmonic nanostructure, the behaviors of linear and nonlinear parts of susceptibility can be coherently controlled. Subsequently, the EIG patterns can be modulated for different distances. For a particular separation distance the linear absorption completely vanishes while the nonlinear refraction intensifies. In this case, the probe energy can be transferred from zero-order to the high orders of diffraction. Such a mechanism for redistributing the probe energy from zero-order to high-order of diffractions stems from the enhancement of cross-Kerr nonlinearity (third order of susceptibility) and vanishing of linear absorption.

## Theoretical model and formulation

**Figure 1:**
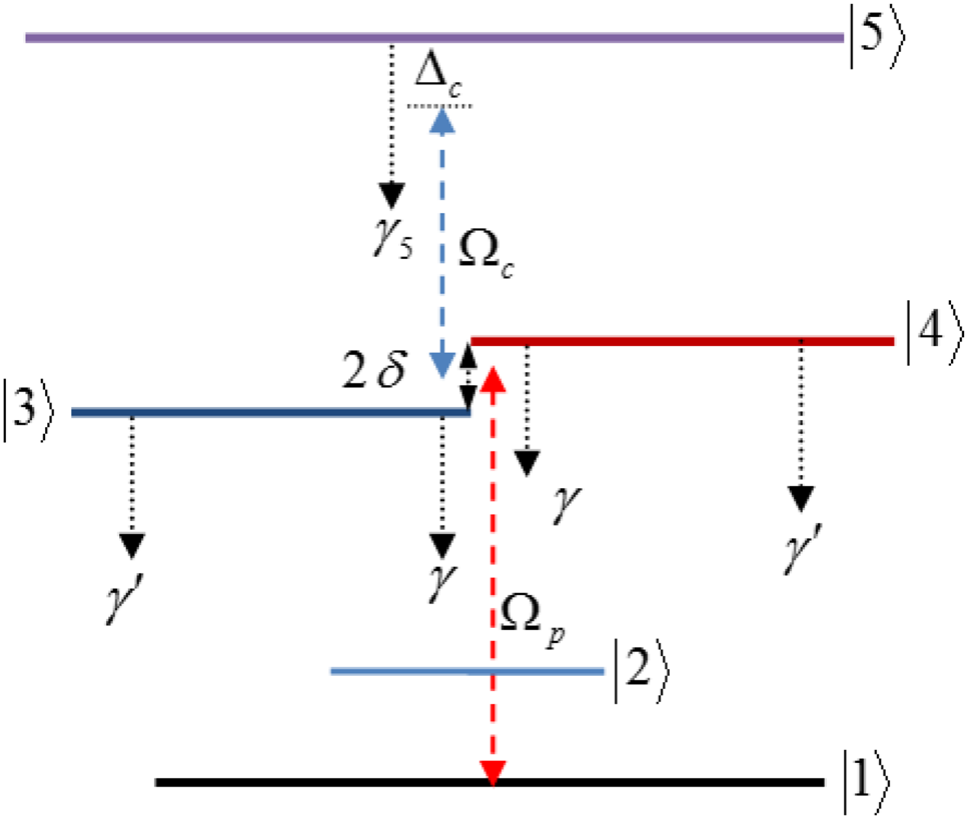
Five-level quantum system interacted with a weak probe and a strong coupling light which is located at distance R from plasmonic nanostructure.

**Figure 2:**
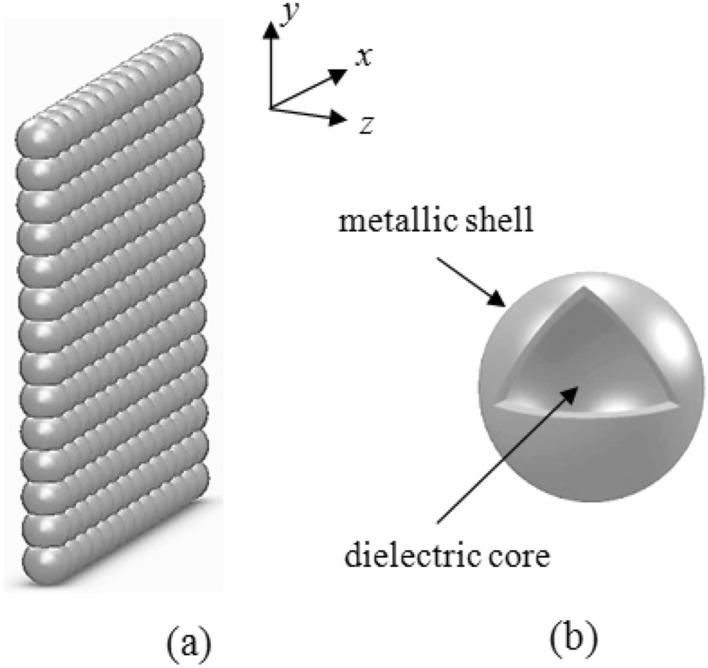
**(a)** Two-dimensional array of nanospheres, **(b)** metal-coated dielectric nanosphere.

The proposed five-level quantum system is displayed in Fig. 1. A weak probe light interacts with the ground state ${\left| 1 \right\rangle}$ and two closely lying middle levels $\left| 3 \right\rangle$ and $\left| 4 \right\rangle$. The levels $\left| 3 \right\rangle$ and $\left| 4 \right\rangle$ are also interacting with an excited level $\left| 5 \right\rangle$. Here, $\Omega_{pi} = {{E_{p} .\mu_{1j} } \mathord{\left/ {\vphantom {{E_{p} .\mu_{1j} } {2\hbar }}} \right. \kern-\nulldelimiterspace} {2\hbar }}$ ($i = 1,2$ and $j = 3,4$) are the Rabi frequencies of the probe field and $\Omega_{ci} ( = {{E_{c} .\mu_{j5} } \mathord{\left/ {\vphantom {{E_{c} .\mu_{j5} } {2\hbar }}} \right. \kern-\nulldelimiterspace} {2\hbar }}$) correspond to the Rabi-frequencies of the coupling field. The quantities $E_{p}$ and $E_{c}$ show the amplitudes of the probe and coupling fields and $\mu_{l\,k}$ denotes the electric-dipole moment of transition $\left| l \right\rangle \leftrightarrow \left| k \right\rangle$. For the sake of simplicity, we take $\Omega_{p2} = \alpha \,\Omega_{p1} = \Omega_{p}$ and $\Omega_{c2} = \beta \,\Omega_{c1} = \Omega_{c}$. The introduced parameters are defined as $\mu_{14} /\mu_{13} = \alpha \,$ and $\mu_{45} /\mu_{35} = \beta$. We assume that the five-level quantum system is placed at distance R from the surface of the plasmonic nanostructure (Fig. 2). In such a situation, the transitions $\left| 3 \right\rangle$ and $\left| 4 \right\rangle$ to $\left| 2 \right\rangle$ lie within the surface-plasmon band of the plasmonic nanostructure, while the transitions $\left| 3 \right\rangle ,\left| 4 \right\rangle$ to $\left| 1 \right\rangle$ and $\left| 5 \right\rangle$ to $\left| 3 \right\rangle ,\left| 4 \right\rangle$ are spectrally far from the surface-plasmon bands and consequently, are not affected by the plasmonic nanostructure. Therefore, the transitions $\left| 3 \right\rangle$ and $\left| 4 \right\rangle$ to $\left| 1 \right\rangle$ and $\left| 5 \right\rangle$ to $\left| 3 \right\rangle$ and $\left| 4 \right\rangle$ of the quantum system interacts with free-space vacuum electromagnetic modes. This model has been well studied in^29,30^. The equations for the matter fields are

1 $$ \begin{gathered} \dot{A}_{1} = i\Omega_{p} A_{3} + i\alpha \,\Omega_{p} A_{4} , \hfill \\ \dot{A}_{3} = [i(\Delta_{p} - \delta ) - (\frac{{\gamma_{3} }}{2})]A_{3} + i\Omega_{p} A_{1} + i\Omega_{c} A_{5} - \eta A_{4} , \hfill \\ \dot{A}_{4} = [i(\Delta_{p} + \delta ) - (\frac{{\gamma_{4} }}{2})]A_{4} + i\alpha \,\Omega_{p} A_{1} + i\beta \,\Omega_{c} A_{5} - \eta A_{3} , \hfill \\ \dot{A}_{5} = [i(\Delta_{p} + \Delta_{c} ) - \frac{{\gamma_{5} }}{2}]A_{5} + i\Omega_{c} A_{3} + i\beta \,\Omega_{c} A_{4} ,\,\,\, \hfill \\ \end{gathered} $$

where $\Delta_{p} = \omega_{p} - (\omega_{31} + \omega_{41} )/2$ and $\Delta_{c} = \omega_{c} - (\omega_{53} + \omega_{54} )/2$ show the detuning of the probe and coupling lights, respectively. Here,$\omega_{p}$ and $\omega_{c}$ denote the frequencies of the applied fields and $\omega_{i\,j\,}$ is the central frequency of transition $\left| i \right\rangle - \left| j \right\rangle$. The frequency difference between states $\left| 3 \right\rangle$ and $\left| 4 \right\rangle$ is denoted by $2\delta$. Here, $\gamma_{3} = \gamma_{4} = \gamma + \gamma^{\prime},\,\,$ where $\gamma$ and $\gamma^{\prime}$ are the decay rates out of states $\left| 3 \right\rangle ,\,\left| 4 \right\rangle$ to $\left| 2 \right\rangle$ and $\left| 1 \right\rangle$, respectively. Moreover, $\Gamma_{5}$ shows the total electron decay rate of the state $\left| 5 \right\rangle$. The cross-coupling $\eta$ between levels $\left| 3 \right\rangle$ and $\left| 4 \right\rangle$ refers to the quantum interference effect in the system coming from the spontaneous emission in a modified anisotropic vacuum.

Following Ref.^28^, the values of parameters $\gamma$ and $\eta$ can be obtained via the electromagnetic Green’s tensor as:

2a $$ \gamma = \frac{{\mu_{0} \mu^{2} \overline{\omega }}}{2\hbar }\,{\text{Im}} [G_{ \bot } (\vec{r},\vec{r};\overline{\omega }) + G_{\parallel } (\vec{r},\vec{r},\overline{\omega })] = \frac{1}{2}(\Gamma_{ \bot } + \Gamma_{\parallel } ),\,\, $$

2b $$ \eta = \frac{{\mu_{0} \mu^{2} \overline{\omega }}}{2\hbar }\,{\text{Im}} [G_{ \bot } (\vec{r},\vec{r};\overline{\omega }) - G_{{_{\parallel } }} (\vec{r},\vec{r},\overline{\omega })] = \frac{1}{2}(\Gamma_{ \bot } - \Gamma_{\parallel } ),\, $$

where $G(\vec{r},\vec{r},\overline{\omega })$ denotes the dyadic electromagnetic Green tensor, $\vec{r}$ displays the position of the quantum system, $\mu_{0}$ is the permeability of vacuum and $\overline{\omega } = (\omega_{4} + \omega_{3} )/2 - \omega_{2}$ , and $\omega_{j}$ represents the energy of level $\left| j \right\rangle$. Moreover,$G_{ \bot } (r,r,\overline{\omega }) = G_{zz} (r,r,\overline{\omega }),\,G_{\parallel } (r,r,\overline{\omega }) = G_{xx} (r,r,\overline{\omega })$ indicates the components of the electromagnetic Green’s tensor where the symbol $\bot (\parallel )$ denotes a dipole oriented normal along the z-axis (parallel, along the x-axis) to the surface of the nanostructure. Therefore, the spontaneous emission rates normal and parallel to the surface are given by

3 $$ \Gamma_{ \bot (\parallel )} = \mu_{0} \mu^{2} \overline{\omega }{\text{Im}} [G_{ \bot (\parallel )} (r,\vec{r};\overline{\omega })]/\hbar .\, $$

When the quantum system is placed in vacuum, $\Gamma_{ \bot } = \Gamma_{\parallel }$ and $\eta = 0$, meaning that no quantum interference occurs in the system^28,30,31,43^.

By solving the coupled amplitude equations (Eq. 1) in the steady state under the weak probe approximation ($\left| {A_{1} } \right|^{2} = 1$), and substituting into the medium polarization^44^

4a $$ P_{p} = \varepsilon_{0} \chi_{p} E_{p} = 2N(\mu_{13} A_{3} A_{1}^{*} + \mu_{14} A_{4} A_{1}^{*} ),\, $$

the probe susceptibility is given by^44^

4b $$ \chi_{p} = - \frac{N\mu }{{\varepsilon_{0} \hbar }}\chi, $$

where $N$ denotes the atomic density, and

5 $$ \chi = - \frac{{\Xi_{5} (\alpha^{2} \Xi_{3} + \Xi_{4} - 2i\alpha \eta ) - (\alpha - \beta )^{2} \Omega_{c}^{2} }}{{\Xi_{5} (\Xi_{3} \Xi_{4} + \eta^{2} ) - \Omega_{c}^{2} (\beta^{2} \Xi_{3} + \Xi_{4} - 2i\beta \eta )}}.\, $$

Here $\Xi_{3} = \Delta_{p} - \delta + i\frac{{\gamma_{3} }}{2},\,\Xi_{4} = \Delta_{p} + \delta + i\frac{{\gamma_{4} }}{2}$, and $\Xi_{5} = \Delta_{p} + \Delta_{c} + i\frac{{\gamma_{5} }}{2}$. Equation (5) has been obtained via deriving the coefficients $A_{i} \,(i = 2,\,3,\,4)$ from Eq. (1) in the steady state limit, and then replacing them into Eq. (4a). We suppose that $N\mu /\varepsilon_{0} \hbar \simeq 1\Gamma_{0}$, where $\Gamma_{0}$ denotes the decay rates of levels $\left| 3 \right\rangle$ and $\left| 4 \right\rangle$ to level $\left| 1 \right\rangle$ in the vacuum.

In what follows we will obtain a set of expressions relating the linear, third-order and fifth-order susceptibilities of the quantum system. The linear and nonlinear responses of the atomic system under applied external fields can be described by a series expansion of Eq. (5). The probe susceptibility $\chi_{p}$ up to the fifth order corresponds to

6 $$ \chi_{p} \simeq \chi^{(1)} + \chi^{(3)} \Omega_{c}^{2} + \chi^{(5)} \Omega_{c}^{4} ,$$

where $\chi^{(1)} ,\chi^{(3)}$ and $\chi^{(5)}$ show the first, third and fifth orders of susceptibility that are achieved as

7a $$ \chi^{(1)} = - \frac{{\alpha^{2} \Xi_{3} + \Xi_{4} - 2\alpha i\eta }}{{(\Xi_{3} \Xi_{4} + \eta^{2} )}}\,, $$

7b $$ \chi^{(3)} = \frac{1}{{\Xi_{5} (\Xi_{3} \Xi_{4} + \eta^{2} )}}[(\alpha - \beta )^{2} + \chi^{(1)} \frac{{(\beta^{2} \Xi_{3} + \Xi_{4} - 2\beta i\eta )}}{{(\Xi_{3} \Xi_{4} + \eta^{2} )}}], $$

7c $$ \chi^{(5)} = \frac{{( - i\eta (\alpha + \beta ) + \alpha \beta \,\Xi_{3} + \Xi_{4} )^{2} (\beta^{2} \Xi_{3} + \Xi_{4} - 2\beta i\eta )^{2} }}{{(\Xi_{3} \Xi_{4} + \eta^{2} )^{3} \Xi_{5}^{2} }}\,. $$

From Eq. (7a), we can find that the linear probe susceptibility vanishes at frequency

8 $$ \Delta_{p} = \pm \frac{{\sqrt {(2\eta + \Gamma )(2\eta - \Gamma )(4\delta^{2} - 4\eta^{2} + \Gamma^{2} )} }}{2(2\eta + \Gamma )}, $$

where $\gamma_{3} = \gamma_{4} = \Gamma .$ For such values of the detuning, the medium becomes transparent to the probe light.

The linear susceptibility is featured in right-hand-side of Eq. (7b) for the third-order nonlinear susceptibility. Under the situation that the linear susceptibility coefficient is zero, the third-order nonlinearity becomes proportional to $(\alpha - \beta )^{2}$. Thus, when $\alpha = - \beta$ an enhanced nonlinearity of the probe light can be obtained accompanied by vanishing linear absorption. In the next section we take $\alpha = 1$ and $\beta = - 1$ satisfying this situation.

### A. Linear and nonlinear properties of quantum system

In what follows, we investigate the linear and nonlinear properties of the quantum system placed near the plasmonic nanostructure when $\alpha = 1$ and $\beta = - 1$ and for different distances between the quantum system and plasmonic nanostructure. The values of parameters $\Gamma_{ \bot }$ and $\Gamma_{\parallel }$ for different distance R are given in Table 1 (based on^45^).

**Table 1:** Values of $\Gamma_{ \bot }$ and $\Gamma_{||}$ for different distance between quantum system and surface of plasmonic nanostructure.

Distance R (nm)	$\Gamma_{ \bot }$($\Gamma_{0}$)	$\Gamma_{\parallel }$($\Gamma_{0}$)
10.4	27.081	0.105
20.8	6.417	0.038
31.2	1.774	0.021
41.6	0.559	0.021
52	0.196	0.026

**Figure 3:**
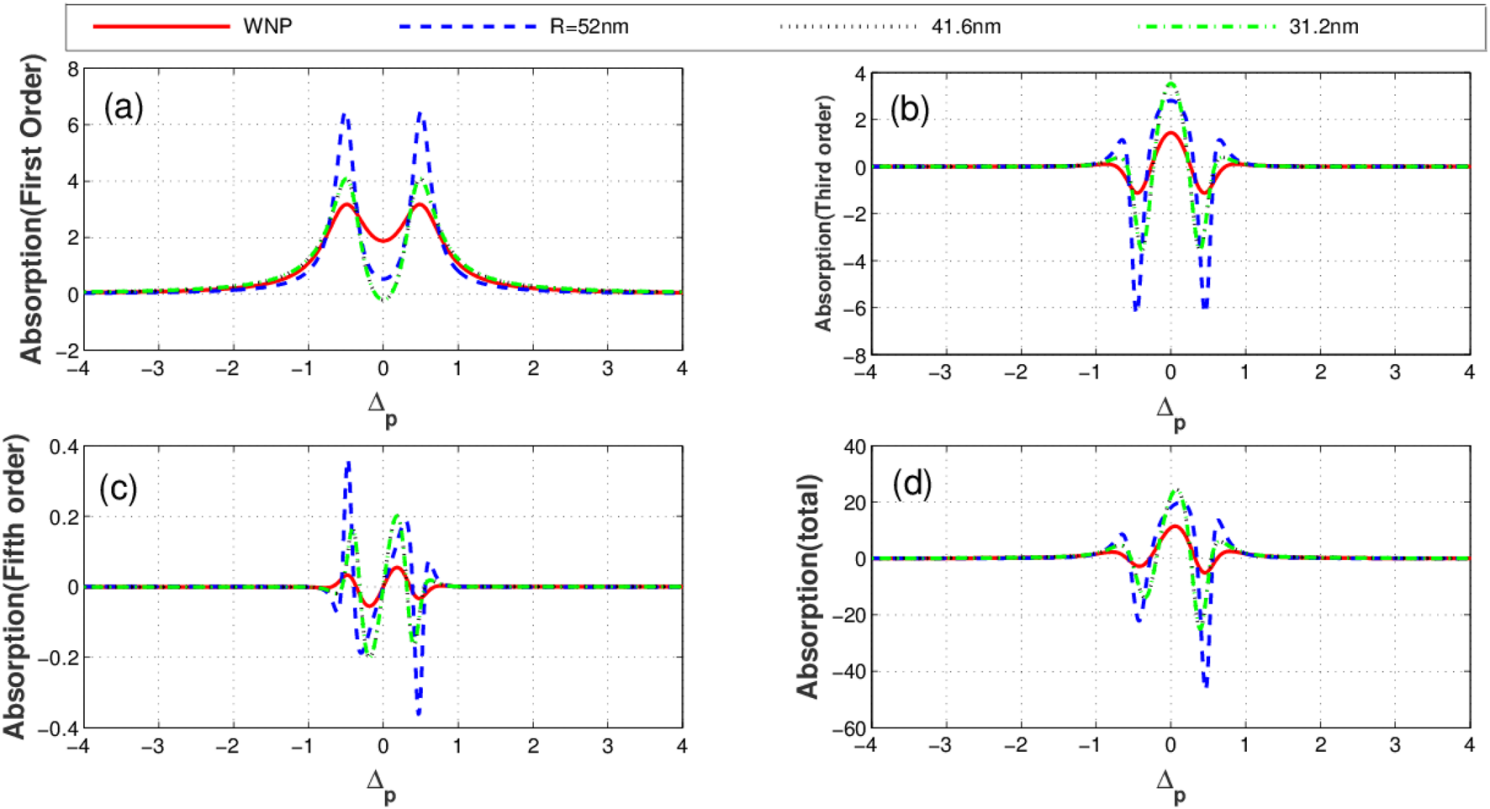
Linear absorption **(a)**, third-order absorption **(b)**, fifth-order absorption **(c)** and total absorption **(d)** plots versus probe detuning for different values of distance between quantum system and plasmonic nanostructure. Solid line corresponds to the case without plasmonic nanostructure, dashed line corresponds to distance R = 52 nm, dotted line corresponds to 41. 6 nm and dotted dashed line corresponds to 31.2 nm. Selected parameters are $\gamma^{\prime} = 0.2\Gamma_{0} ,\,\Delta_{c} = 0$, and $\Omega_{c} = 1\Gamma_{0}$. Our calculation is based on MATLA R2014b software. https://www.mathworks.com/.

**Figure 4:**
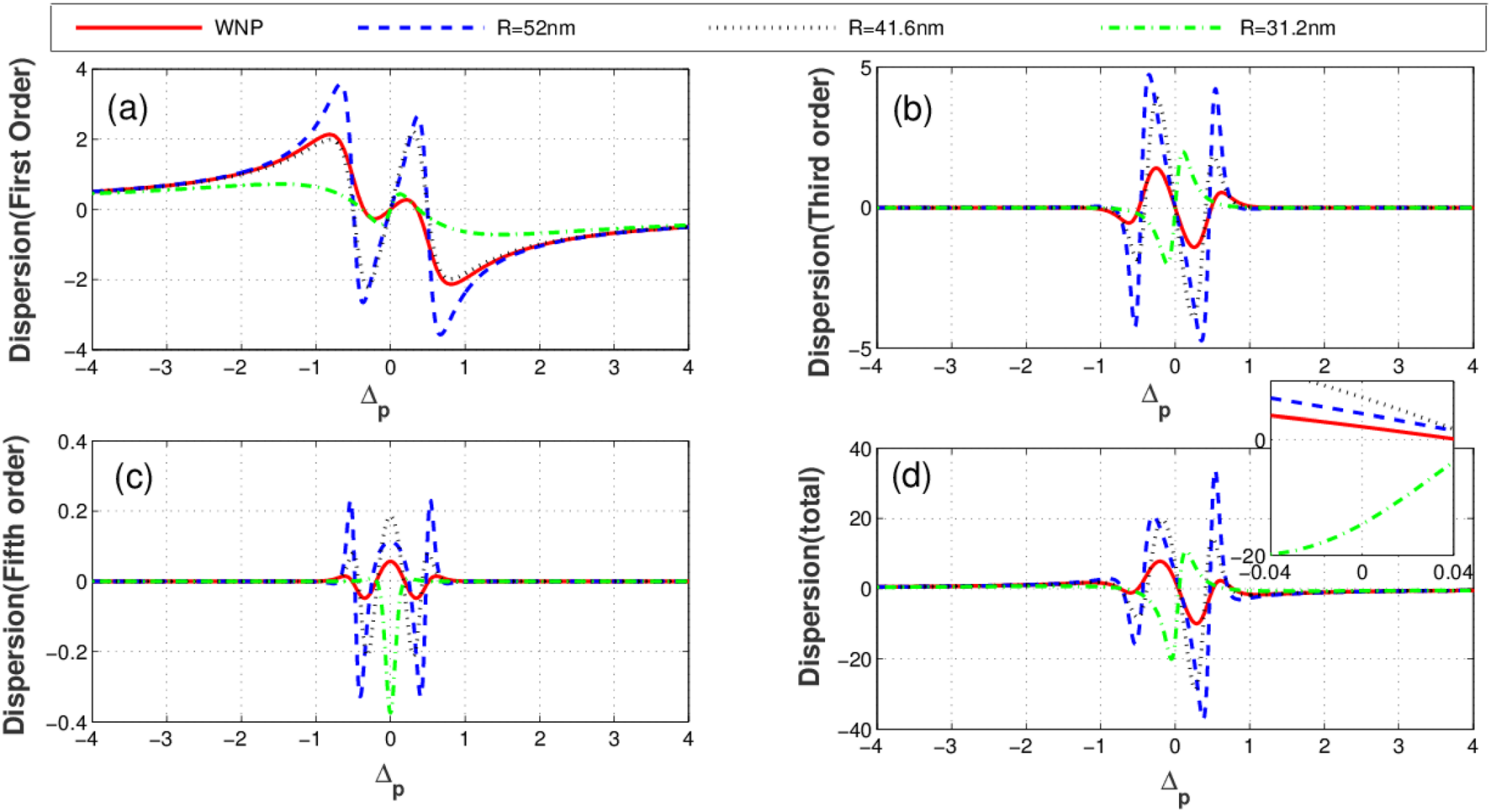
Linear dispersion **(a)**, cross-Kerr nonlinearity **(b)**, fifth-order dispersion **(c) **and total dispersion **(d)** plots versus probe detuning for different values of distance between quantum system and plasmonic nanostructure. Solid line corresponds to case without plasmonic nanostructure, dashed line corresponds to distance R = 52 nm, dotted line corresponds to 41.6 nm and dotted dashed line corresponds to 31.2 nm. Selected parameters are $\gamma^{\prime} = 0.2\Gamma_{0} ,\,\Delta_{c} = 0$, and $\Omega_{c} = 2\Gamma_{0}$. The inset shows the detailed parts of total dispersion at small probe field detuning. Our calculation is based on MATLA R2014b software. https://www.mathworks.com/.

Illustrated in Fig. 3 is the imaginary parts (absorption) of linear (a), third-order (b), fifth-order (c), and total susceptibility (d) versus the probe field detuning for different distances between quantum system and the plasmonic nanostructure. The solid-red plot corresponds to the condition when the quantum system is located at free space (without the plasmonic nanostructure). The blue dashed, black dotted and green dashed-dotted curves represent the corresponding results for the distances 52 nm, 41.6 nm and 31.2 nm between the quantum system and plasmonic nanostructure, respectively. Clearly seen from the figures, the distance (R) of the quantum system from the plasmonic nanostructure has a substantial effect on optical properties of the system. As displayed in Fig. 3a, the linear absorption reduces at $\Delta_{p} = 0$ due to the presence of the plasmonic nanostructure. In contrast, the third-order absorption increases at line center in the presence of the plasmonic nanostructure as shown in Fig. 3b. In Fig. 3c, it is shown how the fifth-order absorption varies by different separations. We observe that the presence of the plasmonic nanostructure leads to the enhancement of the probe field absorption (Fig. 3d). The corresponding linear (a), third-order (cross-Kerr nonlinearity) (b), fifth-order (c) and the total dispersion (d) versus the probe field detuning are plotted in Fig. 4. Clearly, the linear dispersion (Fig. 4a) and cross-Kerr nonlinearity (Fig. 4b) are always zero for the resonant probe field meaning that the plasmonic nanostructure does not affect the resonant linear and third-order dispersion. However, the behavior of the fifth-order dispersion is completely different (Fig. 4c). The largest resonant fifth-order dispersion is obtained for R = 31.2 nm (green dotted-dashed). The total dispersion (Fig. 4d) shows that at $\Delta_{p} = 0$, the dispersion of the probe takes a small but nonzero value. This magnitude changes weakly by altering the distance R. We find that although only the fifth-order dispersion enhances in the presence of the plasmonic nanostructure but it has a minor impact on the total dispersion behavior of the system.

Setting $\Delta_{p} = 0$, Eq. (7a–c), the following analytical expressions are derived for the imaginary and real parts of the probe susceptibility at different orders

11a $$ \left. {{\text{Im}} \,(\chi^{(1)} )} \right|_{{\Delta_{p} = 0}} = \frac{(2\eta - \Gamma )}{{( - \delta^{2} + \eta^{2} - \Gamma^{2} /4)}}\,, $$

11b $$ \left. {{\text{Re}} \,(\chi^{(1)} )} \right|_{{\Delta_{p} = 0}} = 0\,,\,\, $$

11c $$ \left. {{\text{Im}} (\chi^{3} )} \right|_{{\Delta_{p} = 0}} = - \frac{{\frac{{\gamma_{5} }}{2}(4 - {\text{Im}} (\chi^{(1)} )\frac{2\eta + \Gamma }{{ - \delta^{2} - \Gamma^{2} /4 + \eta^{2} }})}}{{(\Delta_{c}^{2} + \gamma_{5}^{2} /4)( - \delta^{2} - \Gamma^{2} /4 + \eta^{2} )}}\,, $$

11d $$ \left. {{\text{Re}} (\chi^{3} )} \right|_{{\Delta_{p} = 0}} = \frac{{\Delta_{c} (4 - {\text{Im}} (\chi^{(1)} )\frac{2\eta + \Gamma }{{ - \delta^{2} - \Gamma^{2} /4 + \eta^{2} }})}}{{(\Delta_{c}^{2} + \gamma_{5}^{2} /4)( - \delta^{2} - \Gamma^{2} /4 + \eta^{2} )}}\,, $$

11e $$ \left. {{\text{Im}} (\chi^{5} )} \right|_{{\Delta_{p} = 0}} = - \frac{{4\delta^{2} \gamma_{5} \Delta_{c} (\Gamma + 2\eta )^{2} }}{{( - \delta^{2} - \Gamma^{2} /4 + \eta^{2} )^{3} ((\Delta_{c}^{2} - \gamma_{5}^{2} /2)^{2} + \gamma_{5}^{2} \Delta_{c}^{2} )}},\,\,\, $$

11f $$ \left. {{\text{Re}} (\chi^{5} )} \right|_{{\Delta_{p} = 0}} = \frac{{4\delta^{2} (\Delta_{c}^{2} - \gamma_{5}^{2} /2)(\Gamma + 2\eta )^{2} }}{{( - \delta^{2} - \Gamma^{2} /4 + \eta^{2} )^{3} ((\Delta_{c}^{2} - \gamma_{5}^{2} /2)^{2} + \gamma_{5}^{2} \Delta_{c}^{2} )}} $$

Such analytical solutions can be advantageous to properly estimate the behavior of the system. The linear dispersion is always zero for the resonant probe light, as clearly seen from Eq. (11b) and Fig. 4a. Also, the resonant cross-Kerr nonlinearity vanishes when $\Delta_{c} = 0$ (see Eq. 11d and Fig. 4b). Yet, the fifth order dispersion exists taking positive and negative values, as illustrated in Eq. (11f and Fig. 4c). In fact, unexpected nonzero behavior of fifth order dispersion can be understood by Eq. (11). For $\Delta_{c} = 0$, both the first-and third order susceptibility vanish as described by Eq. (11b, d), however the values of fifth order susceptibility become nonzero around $\Delta_{p} = 0$. Physically, in two-photon resonance condition the first-and third orders of susceptibility have symmetric behaviors, but fifth-order of susceptibility has asymmetric behavior due to its value at $\Delta_{p} = 0$. Infact, the detuning parameter of the coupling field has essential role in asymmetric properties of the fifth-order of susceptibility.

**Figure 5:**
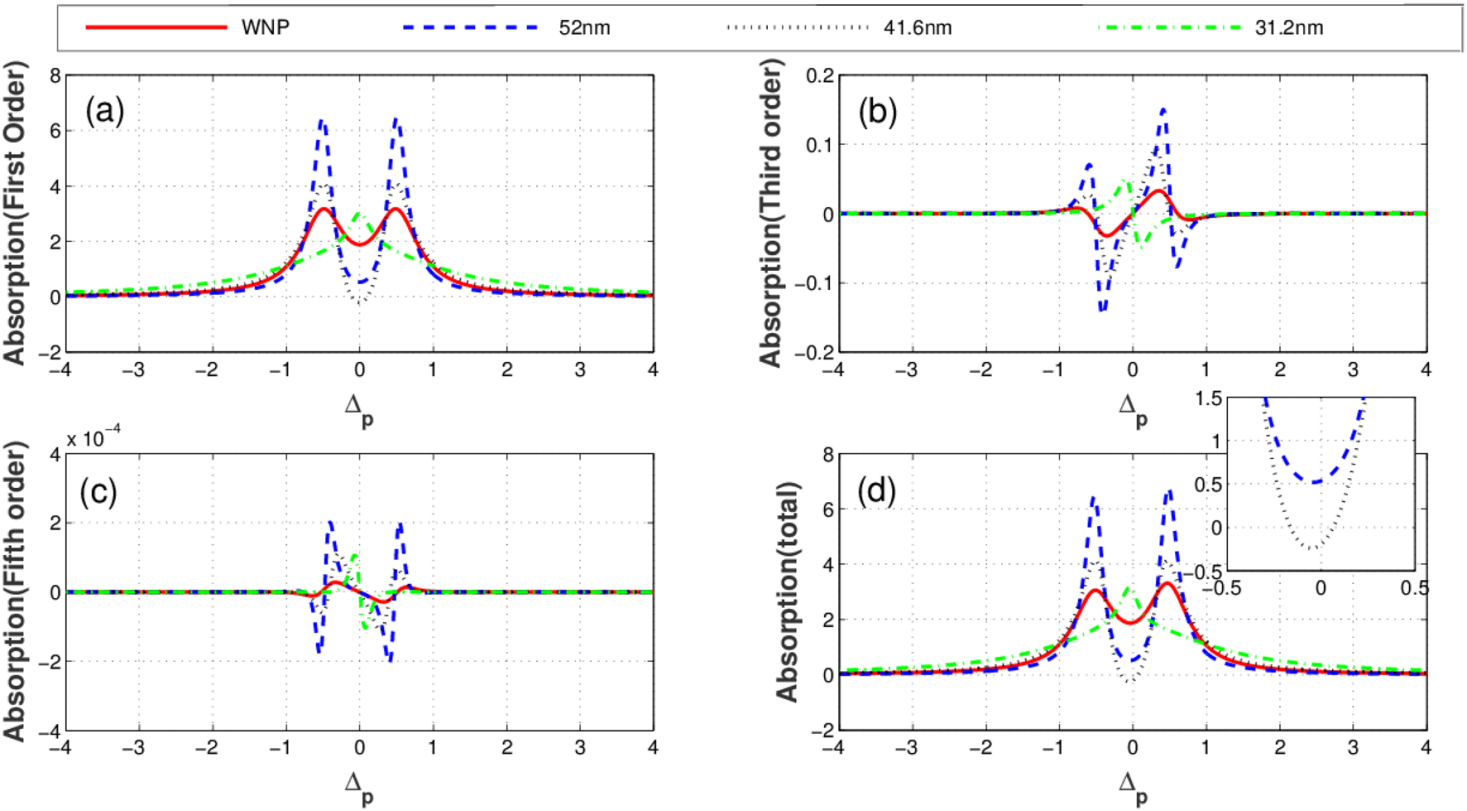
Linear absorption **(a)**, third-order absorption **(b)**, fifth-order absorption **(c)** and total absorption **(d)** plots versus probe detuning for different values of distance between quantum system and plasmonic nanostructure. Solid line corresponds to the case without plasmonic nanostructure, dashed line corresponds to distance R = 52 nm, dotted line corresponds to 41.6 nm and dotted dashed line corresponds to 31.2 nm. Selected parameters are $\gamma^{\prime} = 0.2\Gamma_{0} ,\,\Delta_{c} = - 25\Gamma_{0}$, and $\Omega_{c} = 2\Gamma_{0}$. The inset shows the detailed parts of total absorption at small probe field detuning. Our calculation is based on MATLA R2014b software. https://www.mathworks.com/.

**Figure 6:**
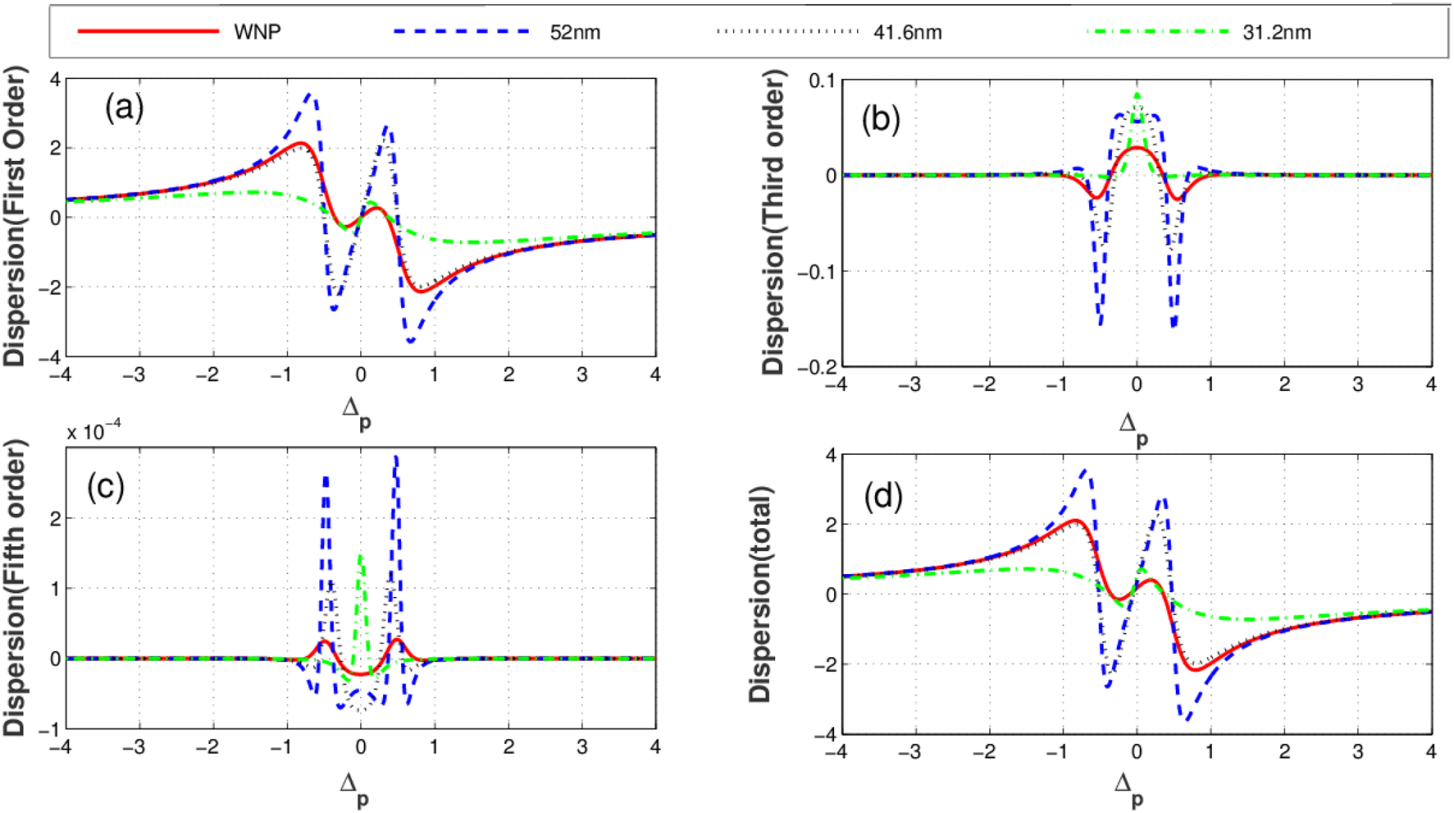
Linear dispersion **(a)**, cross-Kerr nonlinearity **(b)**, fifth-order dispersion **(c)** and total dispersion **(d)** plots versus probe detuning for different values of distance between quantum system and plasmonic nanostructure. Solid line corresponds to the case without plasmonic nanostructure, dashed line corresponds to distance R = 52 nm, dotted line corresponds to 41.6 nm and dotted dashed line corresponds to 31.2 nm. Selected parameters are $\gamma^{\prime} = 0.2\Gamma_{0} ,\,\Delta_{c} = - 25\Gamma_{0}$, and $\Omega_{c} = 2\Gamma_{0}$. Our calculation is based on MATLA R2014b software. https://www.mathworks.com/.

According to Eq. (11d), the cross-Kerr nonlinearity increases when the coupling field is out of the resonance condition with the corresponding transition. Such an enhancement may be accompanied by vanishing linear absorption via adjusting the distance from the plasmonic nanostructure. It is worth noting that the linear absorption is independent of $\Delta_{c}$, according to Eq. (11a). In Fig. 5, we plot the absorption profiles of the different orders of susceptibility versus the probe field detuning for $\Delta_{c} = - 25\,\Gamma_{0}$ and different distances R. As shown in Fig. 5a, the linear absorption exhibits a similar behavior to the case illustrated in Fig. 3a. Panels (b) and (c) of Fig. 5 show the third and fifth-orders of the absorption profile. At $\Delta_{p} = 0$, the nonlinear absorptions are always zero for any distance. However, out of the resonance ($\Delta_{p} \ne 0$) the nonlinear absorption coefficients are non-zero leading to nonlinear absorption or amplification. In such a situation, the probe light is absorbed (solid line) in the absence of the plasmonic nanostructure (Fig. 5d). The total absorption reduces dramatically in presence of plasmonic nanostructure when R = 52 nm (dashed line). Setting R = 41. 6 nm (dotted line), the absorption completely vanishes and the medium becomes transparent. At distance R = 31. 2 nm (green dashed-dotted line), the absorption of the probe light increases. Thus, the linear absorption has a critical role in adjusting the total absorption of the quantum system. Finally, we show different orders of dispersion for different values of R when $\Delta_{c} = - 25\,\Gamma_{0}$ in Fig. 6. Obviously, the linear dispersion (Fig. 6a) behaves the same as in Fig. 4a because it is independent of $\Delta_{c}$. The third (Fig. 6b) and fifth-order dispersion (Fig. 6c) are enhanced on the resonance in the presence of the plasmonic nanostructure. Comparing Fig. 4c with Fig. 6c shows that the giant cross-Kerr nonlinearity is obtained especially for R = 31. 2 nm (green dotted-dashed line). However, the probe field is strongly absorbed in this case. Enhanced cross-Kerr nonlinearity with vanished absorption is observed only for distance and R = 41.6 nm (dotted line). Figure 6d displays how the total dispersion changes with the distance parameter. Particularly, when R = 41. 6 nm (dotted line); the total dispersion reaches to its maximum value around $\Delta_{p} = \pm 0.5\,\Gamma$. In the following section, we will discuss the diffraction patterns of the probe field by adjusting the distance between the quantum system and plasmonic nanostructure, making use of the total susceptibility.

### B. Electromagnetically induced grating

**Figure 7:**
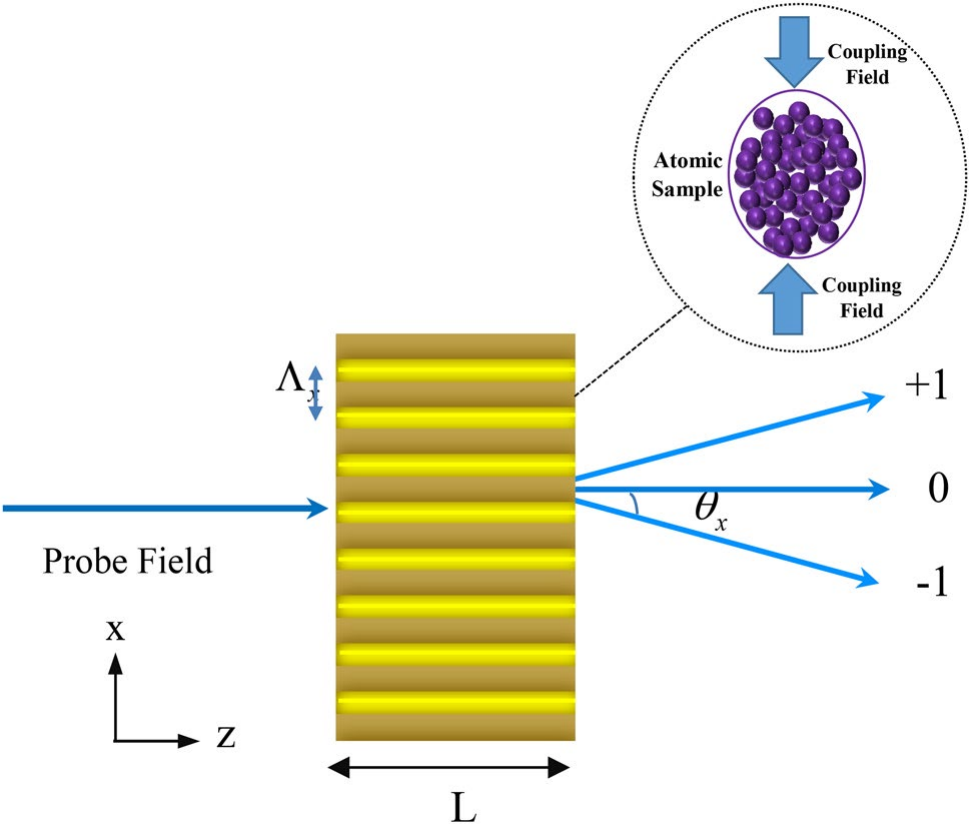
Sketch of the probe and standing-wave coupling fields propagating through the atomic sample.

In recent years, a quantum optical phenomenon known as electromagnetically induced grating (EIG) has attracted much interest because of its remarkable applications. EIG is achieved by replacing the traveling coupling field with a standing-wave field. In such a condition, the optical properties of the quantum system show periodic modulation in space. Such a behavior causes the quantum system takes the role of a grating. As shown in Fig. 7, we assume that the coupling field has a standing-wave pattern given by $\Omega_{c} = \Omega_{c0} \sin ({{\pi x} \mathord{\left/ {\vphantom {{\pi x} {\Lambda_{x} }}} \right. \kern-\nulldelimiterspace} {\Lambda_{x} }})$, with $\Lambda_{x}$ being the spatial frequency of the standing-wave. As indicated by Eq. (6), the linear part of total susceptibility which does not depend on $\Omega_{c}$, has no contribution on the spatial periodic part of the total susceptibility. While, nonlinear parts of susceptibility changes in a spatial period due to the standing-wave coupling field, and consequently the probe field is diffracted into the high-order directions. Note that a large cross-Kerr and nonlinear effects can provide a phase grating with high diffraction efficiencies.

The Maxwell’s equation governs the propagation of the weak probe field through the atomic medium, in the steady state condition and the slowly varying envelop approximation:

12 $$ \frac{{\partial E_{p} }}{\partial z} = ( - \xi + i\,\psi )E_{p} , $$

where $\xi = \frac{2\pi }{\lambda }{\text{Im}} [\chi_{p} ]$ and $\psi = \frac{2\pi }{\lambda }{\text{Re}} [\chi_{p} ]$ denote the dispersion and absorption of the weak probe field, respectively. From Eq. (12), the normalized transmission function can be obtained as

13 $$ T(x) = e^{{ - {\text{Im}} (\chi_{tot} (\omega_{p} ))L}} e^{{i{\text{Re}} (\chi_{tot} (\omega_{p} ))L}} = e^{ - \xi (x)} e^{i\psi (x)L} . $$

**Figure 8:**
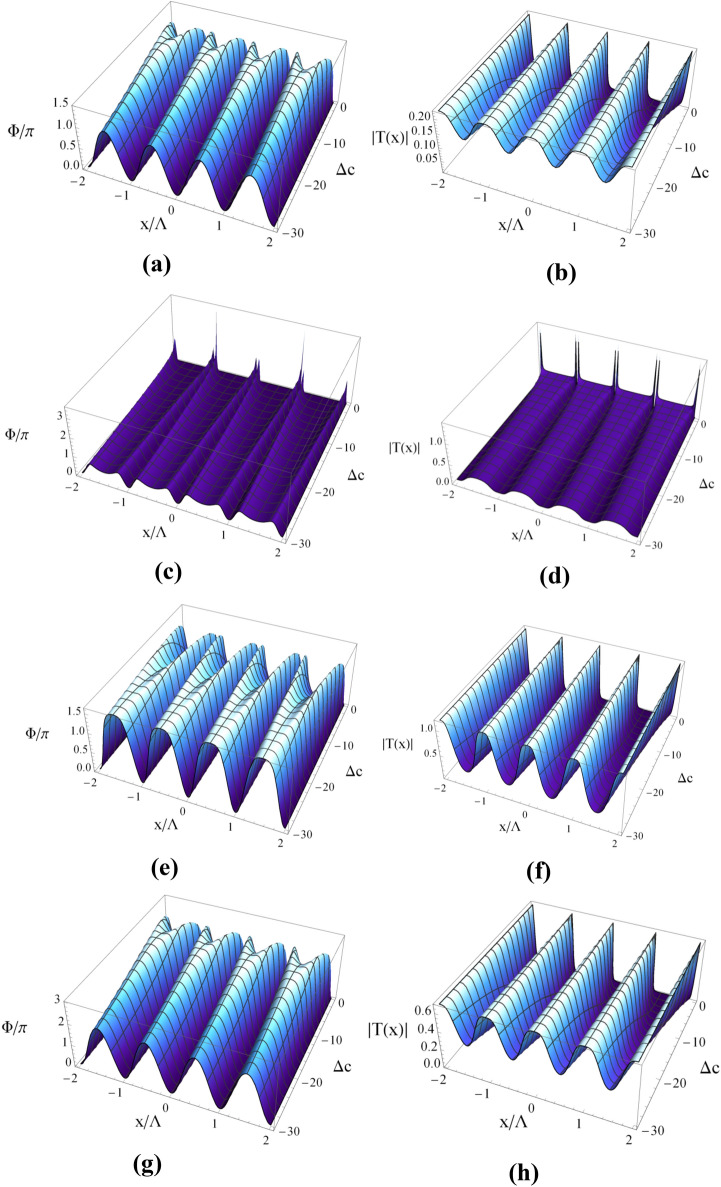
Three dimensional plot of phase (right column) and amplitude modulations (left column) versus x and $\Delta_{c}$ without plasmonic nanostructure **(a)**, at distance R = 31.2 nm **(b)**, distance R = 41.6 nm **(c)** and distance R = 52 nm **(d)**. Selected parameters are same as Fig. 3. Our calculation is based on MATLA R2014b software. https://www.mathworks.com/.

Here $L$ shows the length of the atomic sample that interacts with the probe field in the z direction. The length is considered in units of $({{\hbar \varepsilon_{0} \gamma_{41} } \mathord{\left/ {\vphantom {{\hbar \varepsilon_{0} \gamma_{41} } {k_{p} N\mu_{14}^{2} }}} \right. \kern-\nulldelimiterspace} {k_{p} N\mu_{14}^{2} }})$. By using the Fourier transform of the transmission function, the Fraunhofer diffraction intensity is obtained as^33^:

14 $$ I_{p} (\theta_{x} ) = \left| {E^{1} (\theta_{x} )} \right|^{2} \frac{{\sin^{2} (M\pi \,X\,\sin \theta_{x} )}}{{M^{2} \sin^{2} (\pi \,X\,\sin \theta_{x} )}} $$

where

15 $$ E^{1} (\theta_{x} ) = \int_{0}^{1} {T(x)\exp ( - i2\pi xX\sin \theta_{x} )dx} , $$

denotes the Fraunhofer diffraction of a single slit. Here, $\theta_{x}$ stands for the diffraction angle with respect to the z direction, $X = \Lambda_{x} /\lambda_{p}$ and M is the number of spatial periods of the grating irradiated by the probe beam. The diffraction order n is determined by the grating equation $\sin \theta_{x} = n/X$, where n is the spatial period number of the atomic grating. The diffraction efficiency of the grating is taken to be the ratio of the intensity in the diffracted output to the intensity of the input. Since $I(\theta )$ is normalized such that the intensity of the input probe field is equal to 1, the diffraction efficiency in any diffraction order can be given by the intensity of $I(\theta )$ for that order. In Fig. 8, we display the three-dimensional (3D) plot of amplitude and phase modulations versus $x$ and $\Delta_{c} = 0$. The amplitude and phase patterns exhibit an inhomogeneous distribution over the spatial period of standing wave, which is varied depending on the distance between the quantum system and plasmonic nanostructure. As shown in Fig. 8a, in the absence of plasmonic nanostructure and for the whole range of coupling field detuning, a large portion of the probe field energy is absorbed during the light propagation through the medium. Meanwhile, a good phase modulation is observed by adjusting parameter $\Delta_{c} = 0$ (roughly equivalent to $1.\,5\,\pi$) (Fig. 8b). The 3D plots of amplitude and phase modulations are demonstrated in Fig. 8c, d in which the quantum system is placed at distance R = 31.2 nm from plasmonic nanostructure. We find that the diffracted light is fragile in all orders of direction because of the significant probe absorption in the quantum system. Changing the parameter $\Delta_{c} = 0$ cannot optimize the value of phase modulation. Setting R = 41.6 nm, it is realized that transferring the light energy from the zeroth-order to the first-order can be made possible by tuning the detuning of the coupling field (Fig. 8e, f). For instance, when $\Delta_{c} = - 25\,\Gamma_{0}$ , the phase modulation reaches $1.5\,\pi$ and amplitude modulation approaches unity. In such setting of parameters, significant phase modulation with the low energy loss over the medium can be achieved. When the plasmonic nanostructure is placed at R = 52 nm, a remarkable phase modulation of $\psi = 3\,\pi$ is observed for $\Delta_{c} = 0$ as shown in Fig. 8g, h. The obtained results showing the amplitude and phase modulations of the probe light in Fig. 8 are in conformity with absorption and dispersion profiles presented in Figs. 3, 4, 5.

**Figure 9:**
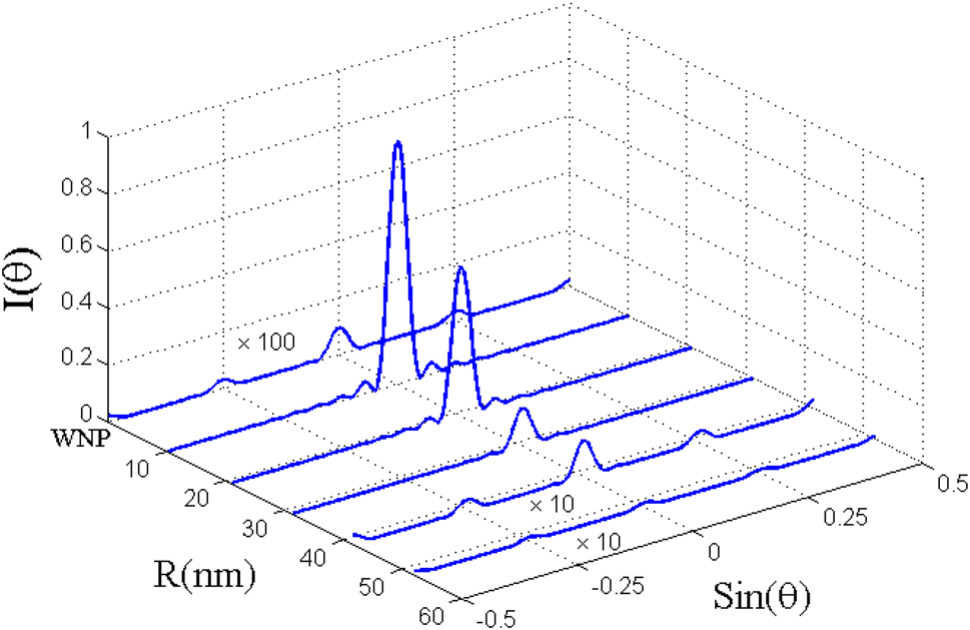
Fraunhofer diffraction pattern versus $\sin \theta_{x}$ and distance R (nm) for $L = 8$ and the selected parameters in Fig. 3. Our calculation is based on MATLA R2014b software. https://www.mathworks.com/.

**Figure 10:**
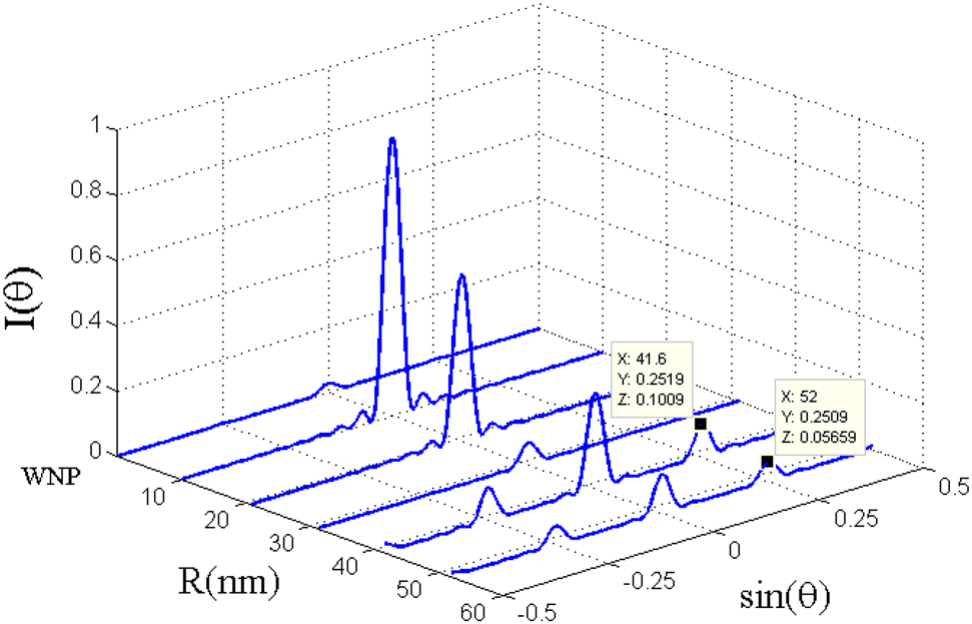
Fraunhofer diffraction pattern versus $\sin \theta_{x}$ and distance R (nm) for $\Delta_{c} = - 25\Gamma_{0}$. Other parameters are same as in Fig. 8. Our calculation is based on MATLA R2014b software. https://www.mathworks.com/.

The corresponding Fraunhofer diffraction patterns versus $\mathrm{sin}({\theta }_{x})$ for different distance parameter R (nm) are illustrated in Fig. 9 for the resonance case $\Delta_{c} = 0$. The total probe susceptibility has been considered in order to study the Fraunhofer diffraction pattern of the probe light. Without the plasmonic nanostructure (WNP), the atomic sample interacting with the standing wave coupling field cannot diffract the probe light in different orders because of the strong absorption. It is clearly seen that in the presence of plasmonic nanostructure at distances R = 10.4 nm, R = 20.8 nm and R = 31.2 nm, the atomic sample behaves completely like an amplitude grating. In this situation, most of the probe energy is gathered in the zeroth-order of diffraction and only a small portion of the energy is transferred to the first-order of diffraction. When R = 41.6 (nm), the phase efficiency of Fraunhofer diffraction increases and some energy is transferred into the first-order. Similar to the case without the plasmonic nanostructure, the probe field is strongly absorbed when the plasmonic nanostructure is placed at R = 52 (nm). Consequently, the atomic system does not behave as a diffraction grating in the presence of the plasmonic nanostructure at such a distance. Finally in Fig. 10, we show the Fraunhofer diffraction patterns versus distance R (nm) and $\mathrm{sin}({\theta }_{x})$ for $\Delta_{c} = - 25\,\Gamma_{0}$. For the non-resonance coupling field, the performance of the presented EIG is improved and the first-order diffraction intensity increases. Note that the behavior of the system in the absence of the plasmonic nanostructure is not affected by the new condition and the probe light is not diffracted due to the strong absorption. In the presence of the plasmonic nanostructure and for larger distances, the ability for the grating is significantly improved, particularly for R = 10.4 (nm). When the quantum system located at the distance R = 41.6 (nm) from the surface of the plasmonic nanostructure, a reasonable portion of probe energy is transferred to the first-order of diffraction. In fact, we are moving to the regime where a considerable portion of energy is transferred from zero-order to first-order of diffraction direction with a high efficiency. This is the distance from the surface of plasmonic nanostructure for which we observed an enhanced cross- Kerr nonlinear with vanishing absorption effects.

## Conclusion

In summary, we have theoretically studied the quantum linear and nonlinear effects in a five-level atom-light coupling placed near a plasmonic nanostructure. The quantum scheme involves a double-V-type subsystem coupling to an excited state by a coupling field. The coupling field can be a position-dependent standing-wave leading to periodic spatial optical patterns. It is found that, by adjusting the distance between the quantum system and plasmonic nanostructure, the linear and nonlinear optical properties of the system can be manipulated by the frequency detuning of coupling light. For a coupling light with standing-wave pattern, we have investigated the Fraunhofer diffraction pattern of the probe light when the quantum system located near the plasmonic nanostructure. It is realized that for the modified nonlinear susceptibility of quantum system, the Fraunhofer diffraction pattern can be created and controlled with different parameters. In the presence of the plasmonic nanostructure, the diffraction efficiency is improved and the first-order diffraction intensity increases when the coupling field is tuned away from resonance. Such a mechanism provides the possibility to switch from an amplitude grating to a phase grating via tuning the distance between the atomic system and nanostructure plasmonic. This model may be applied for optical switching in the optical networking and communication. An optimal distance to achieve suitable nonlinear phenomena has been obtained, being equal to 41. 6 nm.
